# Oral vs parenteral drug administration: Quantifying the future health costs of routine prescribing choices through their environmental scope

**DOI:** 10.1016/j.joclim.2026.100757

**Published:** 2026-09-22

**Authors:** Benjamin de Sainte Marie, Baptiste Verhamme, Camille Carton, Zélie Charuel, Virginie-Eve Lvovschi, Max Piffoux, Manon Roche

**Affiliations:** a Department of Internal Medicine, Centre Hospitalier Universitaire de la Timone, Assistance Publique Hôpitaux de Marseille (AP-HM), France; b Université Paris Cité and Université Sorbonne Paris Nord, Inserm, INRAE, Centre for Research in Epidemiology and Statistics, F-75004 Paris, France; c Emergency Department, Centre Hospitalier Universitaire Hôpital Nord, Marseille, France; d Service central des Opérations Pharmaceutiques, Assistance Publique Hôpitaux de Marseille (AP-HM), France; e Primum Non Nocere agency, Béziers, France; f Emergency Department, Hôpital Edouard Herriot, Hospices Civils de Lyon, Lyon, France; g Laboratory "Research on Healthcare Performance" (RESHAPE), INSERM U1290, Université Claude Bernard Lyon 1, Lyon, France; h Centre Léon Bérard, Lyon, France; i Aix Marseille University, ICR, UMR CNRS 7273, Marseille, France

**Keywords:** Parenteral, Intravenous, Oral, Climate change, DALY, Life cycle analysis

## Abstract

**Background:**

Climate change is a major threat to global health, and healthcare is itself a substantial contributor. Choosing the route of drug administration may represent a simple opportunity to reduce environmental impact without compromising care.

**Objective:**

To quantify and compare the incremental environmental impacts and projected future health losses associated with oral, intravenous (IV), and intramuscular (IM) drug administration.

**Methods:**

We performed a life cycle assessment of route-specific materials and packaging associated with oral and parenteral administration scenarios in the emergency departments of the public hospitals of Marseille, France. The functional unit was administration of 100 mg ketoprofen. The active pharmaceutical ingredient was excluded to isolate the environmental impact attributable to the administration route. Environmental impacts were assessed using ReCiPe 2016 and converted into disability-adjusted life minutes (DALMs).

**Results:**

GHG emissions were: 19 g CO₂e for oral administration, 279 g CO₂e for IM injection, 440 and 521 g CO₂e for IV flash and IV syringe pump administration with a pre-existing catheter respectively, and 776 g CO₂e for IV administration requiring catheter placement. Oral administration also had the lowest impact across all 18 midpoint environmental indicators. Projected health losses ranged from 0.13 to 5.59 DALMs, with climate change accounting for 88–92% of the total impact.

**Conclusions:**

When clinically appropriate, prioritizing oral administration and reducing unnecessary IV use may substantially decrease healthcare-related emissions while maintaining quality of care, representing a simple step toward more sustainable healthcare and contributing to health for future generations.

## Highlights

• Oral administration generated up to 41 × less greenhouse gas emissions than IV administration with a catheter placement.
• Projected health losses ranged from 0.13 DALMs (oral) to 5.59 DALMs (IV with catheter placement).
• Prioritizing oral treatment reduces healthcare-related emissions and contributes to health for future generations.

## 1 Introduction

Climate change is caused by greenhouse gas (GHG) emissions of anthropogenic origin [1]. The healthcare sector is responsible for 8% of GHG emissions in France, with the purchase of medications (29%) and medical devices (21%) accounting for half of these emissions with similar orders of magnitude in most high-income countries [2,3]. According to *The Lancet Countdown* climate change is "the greatest global health threat facing the world in the 21st century, but it is also the greatest opportunity to redefine the social and environmental determinants of health" [4]. Considering these aspects, the healthcare sector is both a stakeholder and a victim of climate change. By taking concrete actions to reduce our environmental impact while maintaining the same or enhanced quality of care, we can mitigate the health effects of climate change, notably including through appropriate prescribing.

Although intravenous (IV) or intramuscular (IM) treatments are sometimes necessary, there are many advantages to using oral treatment, such as: reduced risk of infection, patient comfort, shorter hospital stays, time saved for nurses, and lower costs. There have also been recent concerns on microplastics entering human blood directly through IV injection with potential harm to human health [5,6]. The life cycle impact of choosing the appropriate route of administration has recently been studied for paracetamol, penicillin and morphine, with administration materials and packaging repeatedly identified as major contributors [[7], [8], [9], [10], [11]]. Because the route of administration is a frequent prescribing decision, even small reductions in the environmental burden of individual administrations may translate into substantial cumulative benefits.

It remains difficult to understand for a non-specialist what a kg CO₂e or t CO₂e is worth, especially when it is compared to patients’ health. Some assessment models like ReCiPe 2016 provide factors allowing the conversion of pollutant emissions (e.g. kg CO₂e) into future quality adjusted life years (QALY) or disability adjusted life years (DALY) loss for future generations. Alternatively, these metrics may be converted into disablity adjusted life minutes (DALMs) [12].

The aim of this study was to quantify and compare the incremental environmental impacts associated with oral, IV, and IM drug administration, attributable to route-specific components, together with their associated projected future health losses. We also discuss the clinical trade-offs associated with each route.

## 2 Methods

### 2.1 Life cycle analysis

#### 2.1.1 Scope, functional unit and system boundary

The environmental impact of a single IV infusion (drip and syringe pump), IM, and oral administration in the emergency departments of the public hospitals of Marseille, France, was assessed through a Life Cycle Assessment (LCA) using the ReCiPe 2016 Midpoint and Endpoint methodology, in accordance with ISO 14040 standards [12]. Environmental impacts were modeled using SimaPro software (version 8.0) and the Ecoinvent database (version 3.9.1). Eighteen midpoint environmental indicators were evaluated, including climate change, stratospheric ozone depletion, ionizing radiation, photochemical smog formation (human health and terrestrial ecosystems), fine particulate matter formation, terrestrial acidification, freshwater and marine eutrophication, terrestrial, freshwater and marine ecotoxicity, human carcinogenic and non-carcinogenic toxicity, land use, mineral and fossil resource scarcity, and water consumption.

The entire life cycle ("cradle to grave") of the route-specific components associated with drug administration was considered. The functional unit was a single administration of 100 mg of ketoprofen through five scenarios: oral administration, IM injection, IV administration with a pre-existing catheter, IV syringe pump administration with a pre-existing catheter, or IV administration requiring catheter placement. The system boundary was restricted to the route-specific components (medical devices, consumables, and packaging), whereas the active pharmaceutical ingredient (API) was intentionally excluded to isolate the incremental environmental impact attributable to the route of administration. This approach was considered appropriate because equivalent doses are generally administered across administration routes for many commonly used medications, and previous studies reported minimal differences in API-related GHG emissions between oral and IV paracetamol formulations (<5 g CO₂e) [9,10]. Accordingly, ketoprofen should be regarded as a representative case study for comparing administration routes rather than as the subject of a complete pharmaceutical LCA.

#### 2.1.2 Route-specific components

Route-specific components varied according to the administration route. The oral scenario included the blister packaging and a medicine cup. All parenteral scenarios included alcohol-based hand rub, disinfectant wipes, a ketoprofen glass vial (packaging only), a drawing-up needle, and materials for skin antisepsis or catheter manipulation (sterile gauze and 5% alcoholic povidone-iodine solution) whenever required by the procedure. The IM scenario additionally included a 10 mL syringe, a hypodermic needle, and non-sterile nitrile gloves. The IV syringe pump scenario included a 50 mL syringe, a 100 mL saline bag, infusion tubing, and a syringe pump. The IV scenario with a pre-existing catheter included a 10 mL syringe, a 100 mL saline bag, and infusion tubing. The IV scenario requiring catheter placement included (additionally to the IV scenario with a pre-existing catheter) sterile gloves, a peripheral venous catheter, a transparent dressing, a 10-mL saline syringe for catheter flushing, and an adhesive dressing (Fig. 1).

**Fig. 1:**
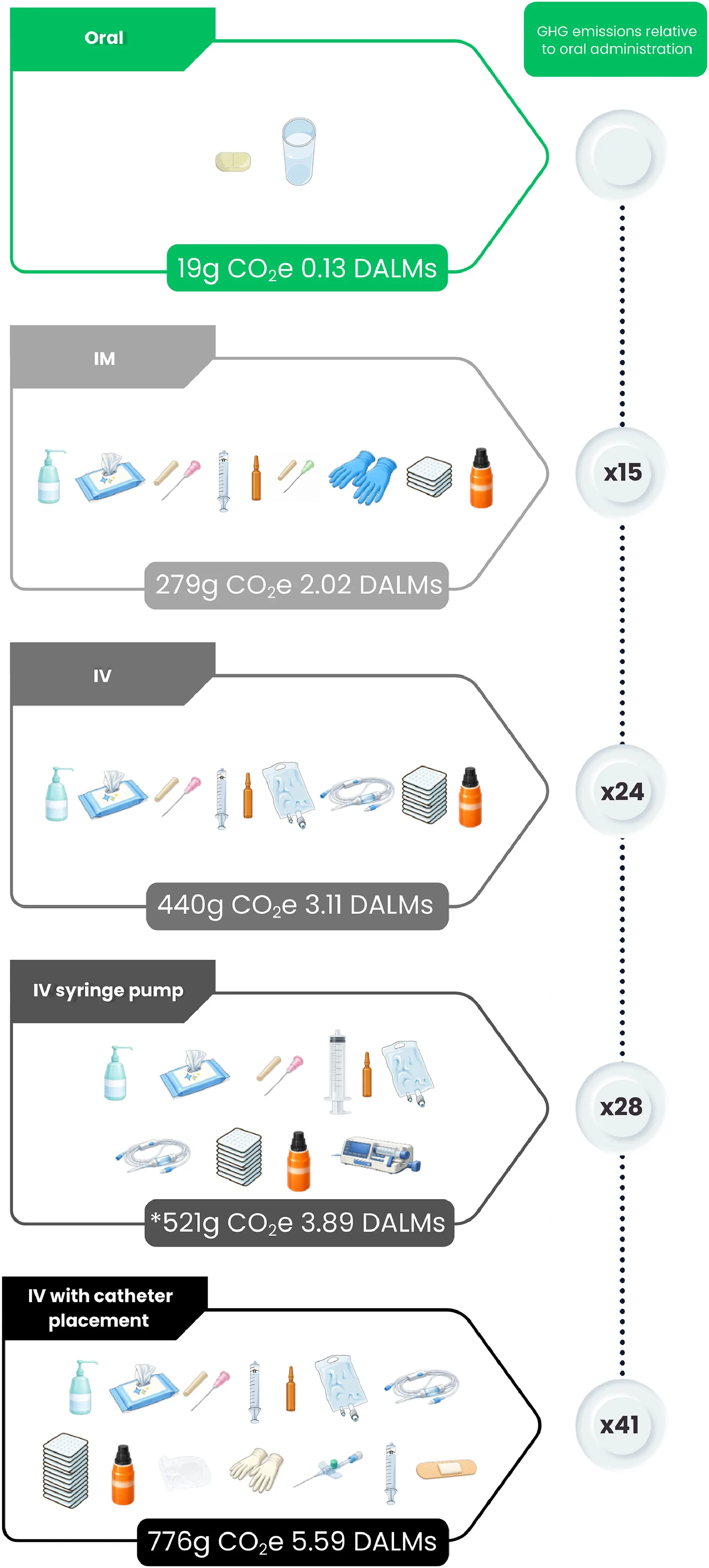
Greenhouse gas emissions and projected future health losses of drug administration routes.

Greenhouse gas (GHG) emissions (gCO2e per administration) and associated projected future health losses expressed as disease-adjusted life minutes (DALMs) are shown for oral, intramuscular (IM), and intravenous (IV) administration, including IV infusion with or without a pre-existing catheter and IV syringe pump administration. Environmental impacts were estimated using life cycle assessment in accordance with ISO 14040 standards using the ReCiPe 2016 methodology. DALMs were derived from midpoint environmental impacts using the egalitarian ReCiPe endpoint perspective, reflecting projected long-term health consequences of emissions within an intergenerational equity framework. Across all scenarios, oral administration was consistently associated with substantially lower environmental impact and projected future health loss compared with parenteral routes. Illustrations are schematic and provided for visual representation of material

#### 2.1.3 Life-cycle inventory

Life-cycle inventory data were compiled for all route-specific components included within the defined system boundary. Data were obtained from manufacturers' technical datasheets and direct communication with suppliers. The inventory included raw material extraction, product manufacturing, material composition, manufacturing location, transport from the manufacturing site to the healthcare facility, product use, and end-of-life waste management. Electricity-related processes were modeled using the geographically appropriate electricity mixes available in the Ecoinvent database whenever possible, according to the country of manufacture or use of each component (e.g. French electricity mix for use of syringe pump).

The following processes were excluded because reliable data were unavailable or their contribution was considered negligible for the comparative assessment: transport of raw materials to manufacturing sites, transport from manufacturing to distribution sites, energy consumption associated with hospital buildings, hospital construction and renovation, and capital equipment (e.g. tables, computers, and notebooks). Water consumption was not assessed for the IM scenario because nitrile polymerization results in a net release of water, and available inventory data do not distinguish clean from grey water.

### 2.2 Conversion from midpoint to endpoint (DALYs) using ReCiPe egalitarian scenario

We estimated the detrimental impact of emissions on future population health by converting midpoint results (amount of GHG and other pollutants) into projected disability-adjusted life years (DALYs), then into disability-adjusted life minutes (DALMs), using the egalitarian perspective of ReCiPe 2016 [12]. This perspective was chosen to reflect the principle of intergenerational equity, whereby future health losses are not discounted relative to present health losses, as discussed by the Intergovernmental Panel on Climate Change (IPCC) and Ducrot et al. [1,13]. The conversion included all available impact pathways and a long-term time horizon (500–1000 years), consistent with the persistence of GHG and other pollutants in the atmosphere [12]. DALMs were used only as an interpretability metric and should not be interpreted as directly observed patient outcomes.

## 3 Results

GHG emissions varied markedly by route of administration. Oral administration emitted 19 g CO₂e per administration, compared with 279 g CO₂e for IM injection, 440 g CO₂e for IV administration with a pre-existing catheter, 521 g CO₂e for IV syringe-pump administration with a pre-existing catheter, and 776 g CO₂e for IV administration requiring catheter placement. In other words, oral administration emitted 15-fold less than IM administration and up to 41-fold less than IV administration requiring catheter placement (Fig. 1).

Oral administration had the lowest impact across all 18 midpoint environmental indicators. For most non-climate indicators, the ranking from lower to higher impact was oral administration, then IM injection, then IV administration with a pre-existing catheter, then IV syringe-pump administration with a pre-existing catheter, and finally IV administration requiring catheter placement. Exceptions were observed for: 1. mineral resource scarcity and strastospheric ozone depletion, for which the IM scenario had the highest impact, mainly because of nitrile glove use 2. Ionizing radiation for which the IV syringe pump scenario had the highest impact. As specified above, water consumption was not evaluated for the IM scenario (Fig. 2). Results are detailed in Table 1.

**Fig. 2:**
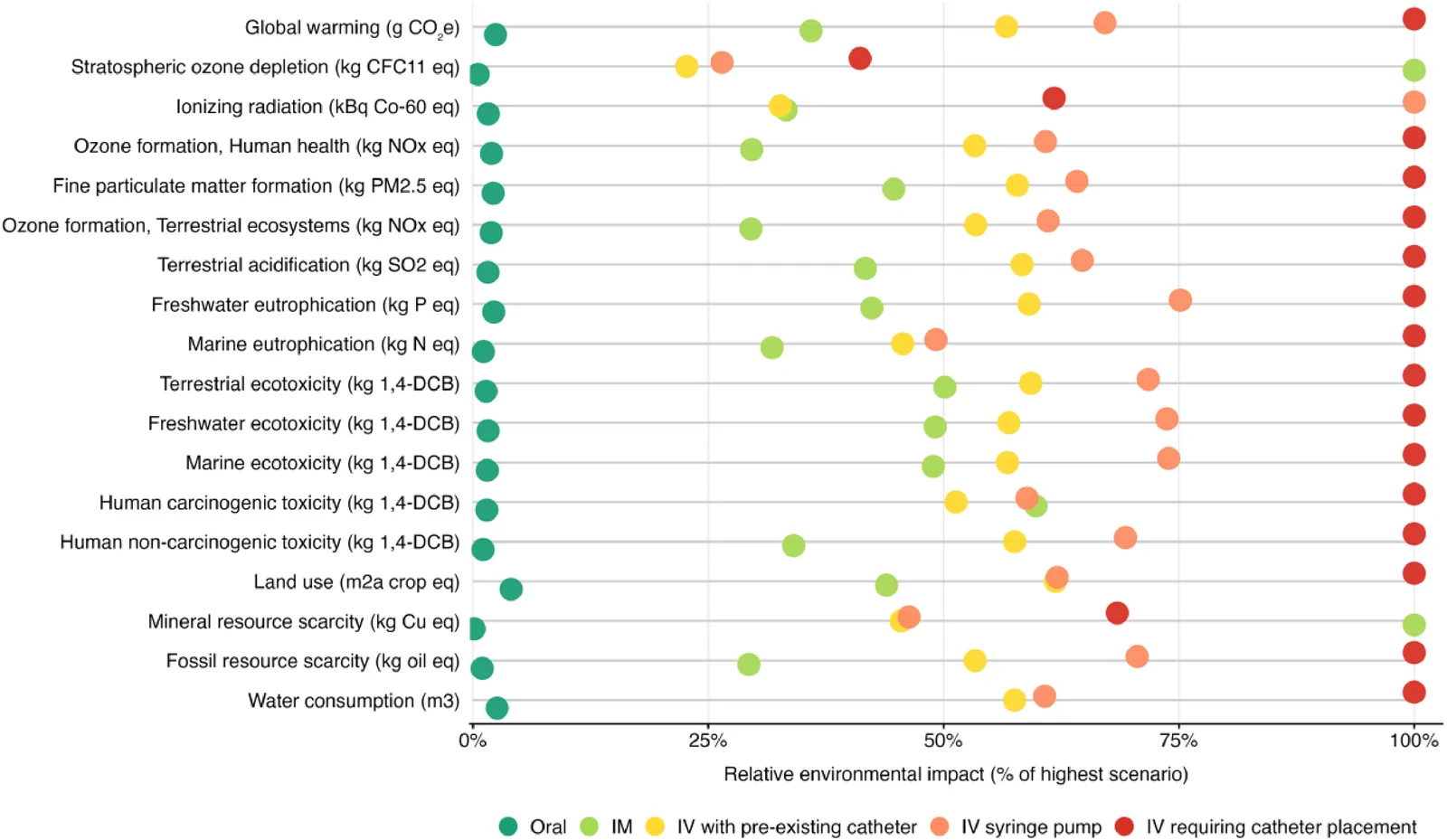
Relative midpoint environmental impacts across drug administration routes.

Values presented in Table 1 were normalized within each midpoint impact category to the highest-impact administration route (100%). Water consumption was not assessed for the IM scenario because nitrile glove production has a negative water balance and available life-cycle inventory data do not distinguish clean from grey water.

**Table 1:** Ecological impact of each scenario for 18 pollutants.

	**Oral**	**IM**	**IV.1**	**Syringe pump**	**IV.2**
Global warming (g CO_2_e)	19	279	440	521	776
Stratospheric ozone depletion (kg CFC11 eq)	8,55E-09	1,58E-06	3,58E-07	4,17E-07	6,49E-07
Ionizing radiation (kBq Co-60 eq)	6,39E-04	1,31E-02	1,29E-02	3,94E-02	2,44E-02
Ozone formation, Human health (kg NOx eq)	4,59E-05	6,90E-04	1,24E-03	1,42E-03	2,33E-03
Fine particulate matter formation (kg PM2.5 eq)	2,69E-05	5,58E-04	7,22E-04	8,01E-04	1,25E-03
Ozone formation, Terrestrial ecosystems (kg NOx eq)	4,70E-05	7,18E-04	1,30E-03	1,48E-03	2,43E-03
Terrestrial acidification (kg SO2 eq)	5,20E-05	1,37E-03	1,92E-03	2,13E-03	3,29E-03
Freshwater eutrophication (kg P eq)	8,60E-06	1,64E-04	2,29E-04	2,92E-04	3,88E-04
Marine eutrophication (kg N eq)	3,41E-06	9,70E-05	1,39E-04	1,50E-04	3,05E-04
Terrestrial ecotoxicity (kg 1,4-DCB)	3,31E-02	1,20E+00	1,41E+00	1,71E+00	2,39E+00
Freshwater ecotoxicity (kg 1,4-DCB)	7,37E-04	2,27E-02	2,63E-02	3,41E-02	4,62E-02
Marine ecotoxicity (kg 1,4-DCB)	9,34E-04	3,02E-02	3,50E-02	4,56E-02	6,17E-02
Human carcinogenic toxicity (kg 1,4-DCB)	5,89E-04	2,41E-02	2,06E-02	2,37E-02	4,02E-02
Human non-carcinogenic toxicity (kg 1,4-DCB)	1,14E-02	3,60E-01	6,08E-01	7,32E-01	1,06E+00
Land use (m2a crop eq)	3,74E-03	4,06E-02	5,71E-02	5,73E-02	9,23E-02
Mineral resource scarcity (kg Cu eq)	3,30E-05	2,58E-02	1,17E-02	1,19E-02	1,76E-02
Fossil resource scarcity (kg oil eq)	3,05E-03	8,99E-02	1,64E-01	2,17E-01	3,07E-01
Water consumption (m3)	2,91E-04	NA	6,51E-03	6,87E-03	1,13E-02

IV.1: patient with a pre-existing catheter; IV.2: patient requiring catheter placement. Water consumption was not assessed for the IM scenario because nitrile polymerization results in a net water release and available life-cycle inventory data do not distinguish clean from grey water.

After conversion into projected future health losses, impacts were 0.13 DALMs for oral administration, 2.02 DALMs for IM injection, 3.11 DALMs for IV administration with a pre-existing catheter, 3.89 DALMs for the IV syringe-pump administration with a pre-existing catheter, and 5.59 DALMs for IV administration requiring catheter placement. Across scenarios, climate change accounted for approximately 88–92% of the total projected health impact.

## 4 Discussion

Our results are consistent with carbon footprint analyses comparing IV and oral administration of drugs in different countries and drug classes. For 1 g of paracetamol, published estimates range from 8.3 g CO₂e to 38 g CO₂e for oral administration and from 310 g CO₂e (plastic vial) to 628 g CO₂e (glass vial) for IV administration when a catheter is already in place [[7], [8], [9]]. Similar conclusions have been reported for oral versus IV penicillin, and packaging has been identified as a substantial contributor to the environmental footprint of morphine [10,11]. To our knowledge, IM and cases where patients have no catheter in place have never been reported. We also analyzed 17 other environmental impacts which follow the same pattern although use of nitrile gloves sets IM as the most polluting scenario for some indicators.

Our analysis assessed the environmental impact of a single administration for each route. However, the environmental impact of a complete treatment course also depends on the number of administrations required, which may differ between routes (e.g. 3 IV injections will have a greater impact than the same treatment delivered as a single continuous IV syringe pump administration). A carbon impact study showed that continuous antibiotic administration versus intermittent administration reduces the carbon footprint by a factor ranging from 2.3 to 6.6, mainly by reducing the number of infusion devices and consumables required [14]. However, the choice between continuous and intermittent administration must first be guided by considerations of active substance stability, bioavailability, and pharmacokinetic properties.

Clinical appropriateness remains the primary determinant of route choice. Although IV administration may achieve faster peak plasma concentrations, this theoretical advantage should be balanced against oral bioavailability and the time required to obtain IV access in patients without a pre-existing catheter. For analgesics, the time to peak plasma concentration ranges from approximately 30 min for paracetamol to 55 min for ketoprofen, and oral bioavailability is usually high in patients without malabsorption (e.g. 85–98% for paracetamol). In a Portuguese oncology surgical ward, nurses spent on average 11.8 min (range 3–50; SD 7.5) for time to vein assessment to successful catheter placement, with a mean of 1.57 puncture attempts (range 1–8, SD ± 1.1) [15]. Therefore, in many routine scenarios, IV or IM administration may offer limited or no clinical advantage over oral administration when oral therapy is feasible and saves nursing time [16,17]. Finally, healthcare expenditure considerations should also be integrated into clinical choices. IV administration is consistently more expensive than oral administration. For example, Davies et al. showed that replacing all IV doses with oral paracetamol tablets would save 98.3% of total direct costs under stable prices [9].

The conversion of midpoint environmental results into DALYs and DALMs is useful because it translates abstract environmental units, such as kg CO₂e or kg 1,4-DCB equivalents, into a health-based metric that is more familiar to clinicians and health technology assessment frameworks. DALMs make the magnitude of projected future health losses more interpretable at the scale of a single prescription decision, while DALYs allow aggregation across populations and comparison with other health impacts. This approach may help incorporate environmental consequences into clinical guidelines, stewardship programs and health technology assessment [18]. However, DALY- and DALM-based projections are not directly observed outcomes; they rely on characterization factors, modeling assumptions, time horizons and value choices, including the chosen egalitarian perspective in our study. They should therefore be interpreted as decision-support metrics rather than precise individual-level predictions.

Several limitations should be acknowledged. First, our analysis should be interpreted as a comparative assessment of the incremental environmental impact attributable to the route of administration rather than a complete life-cycle assessment of pharmaceutical products. Active pharmaceutical ingredients (APIs) were intentionally excluded to isolate the impact of route-specific materials and processes. This assumption is supported by previous work showing minimal differences in API-related GHG emissions between oral and IV formulations, but it may not apply to all drugs, specifically those requiring route-dependent dose adjustments because of differences in bioavailability (e.g. nefopam). Second, the results depend on local products, electricity mix, suppliers, waste pathways and clinical practice, which may affect transferability. Third, some processes were excluded because data were unavailable or considered negligible, including transport between raw-material and distribution stages, hospital building energy and capital equipment. Fourth, endpoint conversion into DALYs and DALMs introduces additional uncertainty. These limitations illustrate the broader lack of transparent, product-level, multi-impact LCA data in healthcare and underline the need for standardized inventories for medicines and medical devices.

Overall, prioritizing oral administration when clinically appropriate and mitigating IV overuse may simultaneously improve patient comfort, reduce costs and workload, and substantially decrease healthcare-related emissions. Although we lack local data on inappropriate IV or IM use when oral administration would be suitable, a recent prospective multicenter study in French emergency departments found that 35% of peripheral intravenous catheters were not clinically indicated, suggesting substantial potential for reducing unnecessary IV use [19]. Ultimately, integrating environmental considerations into routine prescribing should not be understood as a substitute for clinical judgement. Rather, it adds a population-health and intergenerational dimension to decision-making when several clinically appropriate options exist.

## Use of AI

AI tools were used to assist with English language editing, to improve the conciseness of the manuscript and improve images in Fig. 1. The authors reviewed and validated all outputs and take full responsibility for the final content.

## CRediT authorship contribution statement

**Benjamin de Sainte Marie:** Writing – review & editing, Writing – original draft, Formal analysis, Data curation, Conceptualization. **Baptiste Verhamme:** Writing – review & editing, Validation, Methodology, Conceptualization. **Camille Carton:** Investigation, Formal analysis, Data curation. **Zélie Charuel:** Writing – review & editing, Validation, Formal analysis, Data curation. **Virginie-Eve Lvovschi:** Writing – review & editing, Validation, Conceptualization. **Max Piffoux:** Writing – review & editing, Supervision, Data curation, Conceptualization. **Manon Roche:** Writing – review & editing, Supervision, Project administration, Methodology, Funding acquisition, Formal analysis, Conceptualization.

## Declaration of competing interest

The authors declare that they have no known competing financial interests or personal relationships that could have appeared to influence the work reported in this paper.
